# One Size Does Not Fit All: Tailoring Stress Tests for Athletes

**DOI:** 10.7759/cureus.79192

**Published:** 2025-02-17

**Authors:** Priya Patel, David Sanders, Sean Swearingen

**Affiliations:** 1 Cardiology, Rush University Medical Center, Chicago, USA

**Keywords:** athletes, cardiac stress test, cardiovascular health, exercise stress test, supraventricular tachycardia

## Abstract

A 21-year-old male college track athlete presents with exercise-induced tachycardia, reaching up to 240 beats per minute (bpm), accompanied by palpitations and lightheadedness during high-intensity anaerobic exercises such as sprinting. Initial stress testing with an exercise stress echocardiogram was normal and did not induce any arrhythmias. However, an outpatient Holter monitor detected supraventricular tachycardia (SVT), which was induced during sprinting. In this case, by designing a unique stress test protocol that involved mimicking the specific metabolic demands of our athlete’s sport, we were successfully able to elicit symptoms and uncover the arrhythmia. Reliance on standardized exercise stress testing protocols can lead to false reassurance, as they may fail to unmask the patient’s underlying symptoms or cardiac pathology. These protocols do not account for the increased physical fitness of athletes or the physiological differences associated with the "athlete’s heart." This case highlights the need for stress test protocols for athletes to be tailored, incorporating provocative testing that replicates their sport-specific activities or the circumstances surrounding symptom onset.

## Introduction

The patient is a 21-year-old male college track athlete with no significant past medical history who presented with episodes of exercise-induced tachycardia, which he noticed on his Apple Watch, along with associated palpitations, lightheadedness, and occasional chest pain, but no syncope. The episodes are specifically triggered during high-intensity anaerobic exercises, such as sprinting, but not during less intense activities like jogging or weightlifting. He reported four episodes with sudden onset of symptoms, peak heart rates ranging between 200 and 240 beats per minute (bpm), and a duration lasting from one minute to several hours after resting.

Although habitual exercise is known to be beneficial for overall cardiovascular health through the promotion of adaptive cardiac structural and functional remodeling, this does not make competitive athletes immune to cardiovascular pathology. In the case of our patient with symptomatic palpitations, further testing is warranted to rule out potential pathologic causes such as structural heart disease, malignant ventricular arrhythmias, scar-mediated arrhythmias, channelopathies, or other electrical conduction disorders, which could be potentially fatal. 

The term “athlete’s heart” has been used to define the exercise-induced cardiac remodeling that competitive athletes undergo including an increase in left ventricular mass, left ventricular wall thickening, atrial enlargement, ventricular dilation, changes in vasculature, and other features [1]. These adaptive changes help improve exercise capacity by augmenting stroke volume and increasing peripheral vascular resistance [2]. It is important to note that there are slight differences in the varying degrees of these cardiac changes, depending on whether the athletic conditioning involves primarily endurance exercises, as seen in long-distance running or swimming, versus strength exercises, as seen in wrestling or weightlifting [3]. However, studies have shown significant phenotypic overlap among the two types of sports disciplines in part due to the fact that both often require isotonic and isometric exercise training [1,3]. Despite these positive adaptive changes, athletes remain a vulnerable population at increased risk for adverse cardiac events, including sudden cardiac death, especially when performing vigorous physical activities with undiagnosed underlying cardiovascular diseases such as hypertrophic cardiomyopathy, infiltrative or other cardiomyopathies, anomalous coronary arteries, coronary artery disease, electrical system abnormalities, or other pathologies [4,5]. In fact, their increased cardiopulmonary fitness, including higher cardiac output, greater heart rate reserve, and other adaptations, may allow symptoms to remain silent until a life-threatening cardiac event occurs [5,6]. Thorough and accurate testing can provide the athlete with validation that their symptoms are real, reassurance to exercise and compete safely, and education on understanding their limits and when to seek care. Therefore, the development of any symptoms, such as chest pain, dyspnea, palpitations, lightheadedness, or syncope, in an athlete warrants a comprehensive diagnostic workup, not only for safety purposes but also to minimize psychological stress, especially while competing. The following case highlights the challenges posed by traditional stress testing in the unique population of competitive athletes and demonstrates how a more tailored approach is needed to establish a diagnosis, given the inherent differences in each athlete’s underlying cardiopulmonary physiology and the workload capacity required by their respective sports.

## Case presentation

**Figure 1:**
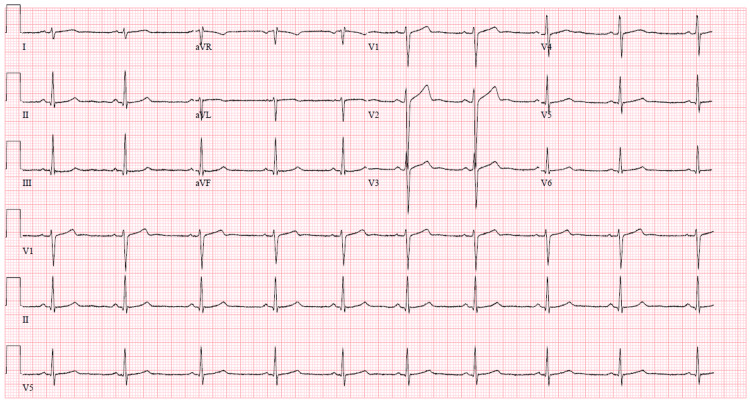
Patient's baseline ECG demonstrating sinus bradycardia, right axis deviation but no evidence of pre-excitation. ECG, electrocardiogram

At the clinic appointment, the patient’s baseline vitals were obtained, including a blood pressure of 119/66 mmHg, heart rate of 64 bpm, weight of 79.4 kilograms, and body mass index of 24.4 kg/m². The initial workup included basic blood tests, such as a complete blood count, comprehensive metabolic panel, high-sensitivity troponin, and thyroid function panel, all of which were within normal limits. A baseline electrocardiogram (ECG) showed no significant abnormalities but noted sinus bradycardia at 58 bpm and right axis deviation, both of which can be normal findings in athletes (Figure 1). An exercise stress echocardiogram was performed, during which the patient exercised for 20 minutes and 20 seconds to Bruce protocol stage 7, reaching a peak heart rate of 196 bpm (98% of the maximal predicted heart rate) and achieving a maximal work rate of 24 metabolic equivalents (METS). The patient did not experience any symptoms, such as palpitations, and no arrhythmias were noted throughout the test. The stress echocardiogram was normal, with no evidence of stress-induced ischemia.

**Figure 2:**
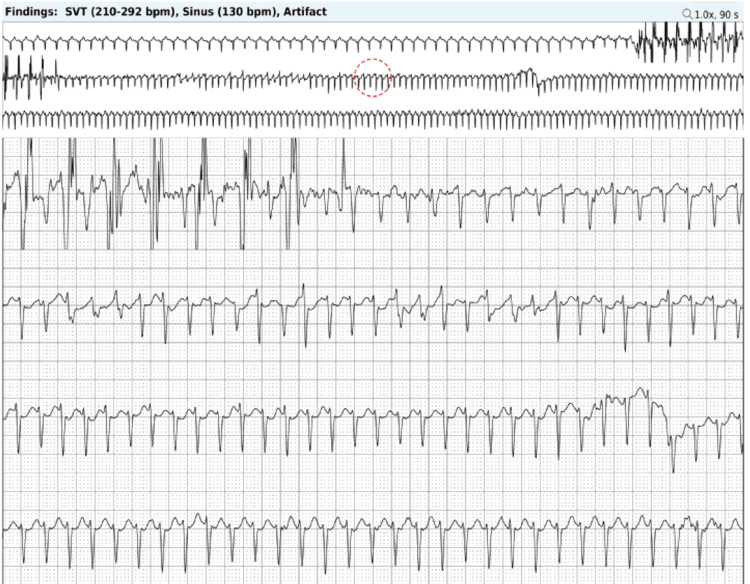
Holter monitor demonstrating SVT. The patient's Holter rhythm monitor demonstrated one episode of SVT that was induced during sprinting with a maximum rate of 292 bpm, average rate of 193 bpm, and lasted one hour and three minutes. SVT, supraventricular tachycardia

As the stress echocardiogram was not able to induce any of his concerning symptoms or arrhythmias, an ambulatory Holter monitor was ordered to more effectively elicit his symptoms and potential arrhythmias by recreating the inciting circumstances. The patient was instructed to wear the Holter monitor and attempt to sprint for a duration long enough to trigger his palpitations and tachycardia. After sprinting for 10 seconds, the patient triggered his monitor as he felt palpitations, which then lasted for an hour before resolving after resting. He had no further symptoms for the remainder of the time he wore the monitor during his regular activities. Upon review, the Holter monitor captured one patient-triggered event, which was an episode of supraventricular tachycardia (SVT) likely caused by atrioventricular nodal reentry tachycardia (AVNRT), given the sudden onset of tachycardia precipitated by a premature atrial contraction. The episode reached a maximum rate of 292 bpm, with an average rate of 193 bpm, and lasted one hour and three minutes (Figure 2). The electrophysiology (EP) team was consulted, and after shared decision-making, medical management of SVT with beta-blockers or other antiarrhythmics was deferred, due to concerns about the potential negative impact on athletic performance, the likelihood of recurrence, and the risk of adverse medication side effects. Instead, the patient elected a more definitive treatment strategy, such as an EP study with ablation, as the cure rate for AVNRT catheter ablation is above 95% [7]. Recurrent SVT can lead to persistent symptoms, psychological stress, decreased athletic performance, impaired quality of life, and potential adverse cardiac effects such as the development of cardiomyopathy [8,9]. The EP study successfully induced AVNRT, after which he underwent a successful ablation of the slow pathway for AVNRT. The patient has now successfully resumed his athletic activities with no restrictions and has not experienced any further symptoms or arrhythmias detected on his Apple Watch. He was advised to follow up in the clinic if his symptoms recur.

## Discussion

This case highlights the importance of performing a comprehensive workup on any symptomatic athlete and tailoring specific exercise stress protocols to the individual metabolic demands of their sport or circumstances that elicited the symptoms. In the case of our track athlete patient with symptomatic palpitations during sprinting, further testing was warranted to rule out potential pathologic electrophysiologic causes such as malignant ventricular arrhythmias, scar-mediated arrhythmias, channelopathies, or other electrical conduction disorders, which could be potentially fatal. 

Although the Bruce protocol is the paradigm for standardized exercise stress testing in the general public, this would not have been an adequate test for our patient due to the many limitations of this testing modality, especially in athletes. One of the major pitfalls of the Bruce protocol is the graded exercise feature involving a considerable ramp-up in speed and elevation with each successive stage equaling a progressive increase in three metabolic equivalents per stage. These considerable jumps in aerobic workload with each stage are designed to elicit symptoms or provoke ischemic changes in a shorter time frame but can often cause uncomfortable leg muscle fatigue [10]. Unfortunately, the Bruce protocol does not account for the increased aerobic capacity and endurance of athletes, which would typically allow them to endure longer stress tests. Instead, athletes often terminate the tests prematurely due to leg fatigue, even before reaching maximum cardiovascular stress [11,12]. In contrast, the modified Balke or Åstrand-Saltin stress treadmill protocols, which keep speed constant but increase elevation with each stage, are preferred alternatives. The more gradual increase in workload, which equates to an increase of one metabolic equivalent per stage, enables athletes to perform more effectively to maximal exhaustion, providing a more accurate reflection of true peak effort [13].

An additional option that providers should be aware of is a dedicated cardiopulmonary exercise (CPX) test, which can better assess a patient’s functional capacity. In addition to the normal parameters acquired during a stress test, CPX will use gas analysis to obtain further physiologic data including maximal oxygen uptake (VO_2_ max), carbon dioxide production (VCO_2_), breath-by-breath analysis of ventilation (VE), respiratory exchange ratio, and anaerobic thresholds, which can provide a rough estimation of whether an individual has truly achieved maximal exertion [14]. Although this test may not perfectly mimic a patient's specific sport, prior data demonstrates that there is a significant increase in catecholamines once a patient reaches their anaerobic threshold during exercise [15]. This test can also be modified to include blood sampling, which can assess oxygen, carbon dioxide, and lactate concentrations at varying intensity levels of exercise [16]. These additional metrics can help guide patients, especially athletes, by providing specific targets to aim for during prolonged aerobic activities such as marathon running or triathlons [17]. In situations where recreating a patient's sport in a monitored environment may be difficult or unfeasible, a cardiopulmonary stress test can serve as a useful alternative to traditional exercise stress testing protocols. It provides an exercise prescription that indicates the point at which the patient reaches their anaerobic threshold and true maximal exertion.

However, in the case of our patient, none of these exercise stress test options were likely to elicit the symptoms or arrhythmia as they involve incremental increases in workload and fail to recreate the physiologic stress and hemodynamic demands that occur when going from rest to sudden bursts of high-intensity exercise such as sprinting. Instead, the approach should focus on attempting to electrocardiographically capture the arrhythmia through ambulatory rhythm monitoring or provocative exercise testing. For our patient, he was instructed to wear a heart rhythm monitor and then mimic his typical race including the sprinting portions that previously brought on his symptoms. Designing a tailored stress testing protocol often takes creativity on the part of the physician to try and simulate the differing aerobic and anaerobic physiologic workloads unique to each athlete or sport. Potential applications of this method have been suggested, including having hockey players wear rollerblades and a safety harness while on a treadmill, having basketball players incorporate box jumps during exercise testing, or having cyclists use a recumbent bike [10]. However, it is important to recognize the limitations in the feasibility of applying these practices, as they would require specialized equipment, physicians who are comfortable with these alternative protocols, and additional training for ancillary staff. Nonetheless, this represents an area for future growth, especially at centers with dedicated sports medicine programs that are better equipped to facilitate this specialized testing.

## Conclusions

Although athletes undergo adaptive cardiac remodeling, which can be beneficial, the development of symptoms always warrants a comprehensive evaluation as they are not completely immune to adverse cardiac pathology. Failure to appropriately design a stress test for the individual athlete can lead to false reassurance for both the athlete and the physician if the suboptimal stress testing method is not able to effectively unmask the true cardiac pathology that may be causing the patient’s symptoms. In the long run, this can lead to increased psychological stress, impaired quality of life due to recurrent untreated symptoms, and an increased risk of adverse events from continuing to exercise with undiagnosed underlying cardiovascular disease. Instead, physicians should tailor exercise stress protocols or provocative testing for athletes to recreate the circumstances that trigger their symptoms or replicate the physiological demands of their sport, thereby increasing the likelihood of diagnosing the underlying cardiac pathology.
